# Editorial: Enteric inflammation and chronic diseases: Is there a link?

**DOI:** 10.3389/fphar.2022.1018144

**Published:** 2022-10-13

**Authors:** Stefania Marzocco, Giuseppe Esposito, Emanuela Esposito, Sherif T. S. Hassan

**Affiliations:** ^1^ Department of Pharmacy, University of Salerno, Fisciano, Salerno, Italy; ^2^ Department of Physiology and Pharmacology “V. Erspamer”, Sapienza University of Rome, Rome, Italy; ^3^ Department of Chemical, Biological, Pharmaceutical and Environmental Sciences, University of Messina, Messina, Italy; ^4^ Department of Applied Ecology, Faculty of Environmental Sciences, Czech University of Life Sciences Prague, Prague, Czechia

**Keywords:** enteric inflammation, chronic disease, intestinal homeostasis, inflammatory markers, metabolic inflammation

The prevalence of chronic diseases has become one of the most significant global health threats of the 21st century. These conditions have a significant toll on people’s quality of life and create both societal and economic burdens. Inflammation is a normal biological defense against infection and tissue damage. Under normal circumstances, the inflammatory response quickly ends after the clearance of infection and injurious agents. However, when inflammation becomes self-perpetuating, it can result in chronic or long-term inflammation and trigger inflammation-mediated damage that contributes to the pathogenesis of several chronic diseases. Growing evidence suggests a close link between inflammation and many chronic health conditions (Germolec et al., 2018). Tackling this problem requires the study of the underlying mechanisms and the development of new drugs and therapeutic approaches.

The articles contributed to this Research Topic cover different aspects related to inflammatory-based diseases by highlighting the underlying mechanisms and diverse pharmacological strategies. Intestinal chronic inflammations, including inflammatory bowel disease (IBD), are among the diseases showing more pronounced increases. These diseases are widespread and impose burdens on healthcare systems worldwide. Experimental studies have also revealed the link between these conditions and other systemic diseases. As the pharmacological options to treat these conditions are limited, research to identify new therapeutic strategies is of great interest. In this Research Topic, Jiang et al. reported the potential activity of Gaudichaudione H, a natural compound isolated from the plant *Garcinia oligantha* Mer., in inhibiting the inflammatory response in macrophages and dextran sodium sulfate (DSS)-induced colitis in mice mainly through the modulation of nuclear factor-κB (NF-κB) and mitogen-activated protein kinase (MAPK) pathways, suggesting its potential application in IBD and macrophage-related inflammatory disease in general. da Rocha et al. reported the results of a combined *in vitro* and *in vivo* study to explore the modulation effect of pioglitazone, a PPARγ ligand, on IBD inflammation. They demonstrated the pivotal role of Annexin A1 (AnxA1), a glucocorticoid-modulated anti-inflammatory protein, as a mediating factor for the therapeutic properties of pioglitazone against IBD. Moreover, the *in vitro* data from macrophages indicated that the therapeutic action of pioglitazone depends on extracellular signal-regulated kinase (ERK) phosphorylation, which involves AnxA1. Intestinal barrier homeostasis is involved in many diseases at different levels, including the pathogenesis of recurrent urinary tract infections. Stepanova’s mini-review article highlights the importance of the cross-talk between intestinal barrier dysfunction and the recurrence of urinary tract infection to guide experimental and controlled studies to clarify the mechanisms underlying the interaction between these conditions. Chronic low-grade inflammation has also been associated with metabolic disorders such as insulin resistance triggered by the excessive production of leukotriene B4 (LTB4), a potent inflammatory lipid mediator, in combination with its receptor BLT1. In this context, Gong et al. examined *in vitro* the effect of berberine on the LTB4–BLT1 axis in the development of inflammation and insulin resistance. Their results suggested that berberine might interfere with BLT1 and alter the LTB4–BLT1 axis, leading to decreased insulin resistance and inflammation. Clinically, septic liver injury is associated with severe systemic inflammation and multiple organ dysfunction syndromes. Yu et al. investigated the pharmacological effect of dexmedetomidine (DEX), a highly selective α_2_-adrenoreceptor agonist, against septic liver injury. Their results suggested that DEX might protect against cecal ligation and puncture (CLP)-induced liver injury, highlighting a novel mechanism in which DEX improves autophagy, which reduces the inflammatory responses in CLP-induced liver injury by regulating the adenosine monophosphate-activated protein kinase/Sirtuin 1 (AMPK/SIRT1) signaling pathway.

Finally, the Guest Editors thank all the authors who have contributed to this Research Topic and provided interesting ideas, approaches, and results, which offer a platform upon which other researchers can build.
